# Factors that contribute to faecal cyclooxygenase-2 mRNA expression in subjects with colorectal cancer

**DOI:** 10.1038/sj.bjc.6605564

**Published:** 2010-02-09

**Authors:** Y Hamaya, K Yoshida, T Takai, M Ikuma, A Hishida, S Kanaoka

**Affiliations:** 1 Department of Molecular Diagnosis, Hamamatsu University School of Medicine, 1-20-1 Handayama, Higashi-ku, Hamamatsu 431-3192, Japan; 2 First Department of Medicine, Hamamatsu University School of Medicine, 1-20-1 Handayama, Higashi-ku, Hamamatsu 431-3192, Japan; 3 Department of Gastroenterology, Hamamatsu Medical Center, 328 Tomitsuka-cho, Naka-ku, Hamamatsu 453-7111, Japan

**Keywords:** colorectal cancer, faeces, RNA, COX-2, exfoliated cell

## Abstract

**Background:**

We previously reported that a faecal cyclooxygenase-2 (COX-2) mRNA assay was useful for identifying colorectal cancer (CRC). This study sought to investigate the factors that contribute to faecal COX-2 mRNA expression in subjects with CRC.

**Methods:**

The study cohort comprised 78 patients with CRC and 36 control subjects. The expressions of COX-2, *β*-2-microglobulin (B2M), carcinoembryonic antigen (CEA), E-cadherin (E-cad), and CD45 mRNA in faeces and COX-2 mRNA expression in tissue were determined by quantitative real-time RT–PCR.

**Results:**

The level of faecal expression of COX-2 mRNA in CRC was significantly higher than that in controls. A significant correlation was found between faecal COX-2 mRNA expression and faecal B2M, CEA, E-cad, or CD45 mRNAs, markers of exfoliated total cells, colonocytes, and leukocytes, respectively. A significant correlation was found between the expression of COX-2 mRNA in faeces and tumour surface area, COX-2 mRNA expression in primary tumour. There was no difference in faecal COX-2 mRNA expression between proximal CRC and distal CRC.

**Conclusion:**

COX-2 mRNA expression in faeces seems to originate from tumour lesion and to be affected by factors such as the number of exfoliated cells, exfoliation of inflammatory cells, COX-2 mRNA expression in tumour, and tumour size.

Colorectal cancer (CRC) is one of the most common causes of cancer-related mortality worldwide. Effective screening to detect cancer is expected to reduce the mortality rate of CRC (Mandel *et al*, 1993; Hardcastle *et al*, 1996; Scholefield *et al*, 2002; Lindholm *et al*, 2008). Faecal occult blood test (FOBT) is a non-invasive and simple test reported to reduce the morbidity and mortality associated with CRC (Mandel *et al*, 1993; Hardcastle *et al*, 1996; Scholefield *et al*, 2002; Lindholm *et al*, 2008). However, the efficacy of FOBT is limited because of the common occurrence of occult bleeding from non-neoplastic sources (Osborn and Ahlquist, 2005) and less sensitivity for proximal CRC (Morikawa *et al*, 2005).

Novel methods are now under development for detection of CRC, including those based on the detection of mutated DNA, DNA integrity (Imperiale *et al*, 2004; Ahlquist *et al*, 2008), cancer-related methylation (Chen *et al*, 2005; Glockner *et al*, 2009), and upregulation of cancer-related gene expression in faecal samples (Kanaoka *et al*, 2004; Koga *et al*, 2008; Yu *et al*, 2008; Takai *et al*, 2009). We previously reported that faecal cyclooxygenase-2 (COX-2) mRNA assay is a sensitive and specific method to detect CRC and adenoma (Kanaoka *et al*, 2004, 2007; Takai *et al*, 2009). However, factors that contribute to faecal COX-2 mRNA expression were not well evaluated. COX-2 mRNA expression levels in faeces may be affected by both the number of exfoliated cells that express COX-2 mRNA and the mRNA degradation in faecal samples. Colonocytes and inflammatory cells are reported to be abundant in the mucocellular layer covering CRC tissue (Ahlquist *et al*, 2000). Some of the colonocytes in this mucocellular layer in CRC were reported to retain tumour-associated antigen (Ahlquist *et al*, 2000), suggesting that some of them may express COX-2 mRNA. Inflammatory cells are known to express COX-2 mRNA (Sano *et al*, 1995). Inflammatory cell infiltrates are commonly seen surrounding and/or infiltrating primary tumours. Therefore, it is possible that the degree of inflammatory cell infiltration influences COX-2 mRNA expression in faeces. The increase in faecal COX-2 mRNA expression may be caused by the increased number of exfoliated cells. Cell exfoliation from colonic epithelium was known to be dramatically increased in neoplasia (Ahlquist *et al*, 2000; Loktionov, 2007). It is well known that COX-2 is overexpressed in >80% of CRCs (Kawasaki *et al*, 2008). The difference in COX-2 mRNA expression among colorectal tumours may affect COX-2 mRNA expression in faeces. Tumour size can be another factor that affects faecal COX-2 mRNA expression through the increase in exfoliated cells and through modification of COX-2 mRNA expression in CRC (Fujita *et al*, 1998).

Faecal COX-2 mRNA expression may be affected by the location of tumour, if the degradation of COX-2 mRNA in the colon is affected by the transit time of stool in the colon.

All these factors, including the number of exfoliating cells, inflammatory cell infiltration, the expression of COX-2 mRNA in cancer tissue, and the size and location of the tumour, may contribute to the expression of faecal COX-2 mRNA in CRC.

In this study, we evaluated the number of exfoliated cells by measuring the faecal expression of β-2-microglobulin (B2M) mRNA that is expressed at similar levels in both normal and cancer cells (Dydensborg *et al*, 2006), and carcinoembryonic antigen (CEA) and E-cadherin (E-cad) mRNAs that are expressed in normal and CRC colonic epithelium (Benchimol *et al*, 1989; Dorudi *et al*, 1993). CD45 mRNA, as a marker of the number of exfoliated inflammatory cells (Charbonneau *et al*, 1988), and the expression of COX-2 mRNA in tumour were also evaluated.

## Materials and methods

### Patients and samples

The institutional local genetic research ethics committee of Hamamatsu University School of Medicine (Hamamatsu, Japan) approved this study. All patients and subjects provided oral and written informed consent: 78 patients with CRC who were diagnosed colonoscopically and histologically, and 36 control subjects with no pathological findings. Stool samples were collected between 2 and 4 weeks after the diagnostic colonoscopy and biopsies, and before surgery or endoscopic resection. Collected samples for RNA isolation were initially stored at 4°C and then transferred to −80°C within 24 h, where they were kept for up to 2 years. Genomic DNA was isolated from the blood of a control subject. The median age of cancer patients and control subjects was 68 years (range, 48–86 years) and 65 years (range, 20–87 years), respectively. Table 1 details the patient profiles. CRC was classified according to the International Union Against Cancer tumour-node-metastasis classification (Sobin and Wittekind, 2002). Cancer patients were classified as proximal colon cancer (caecal, ascending, and transverse colon cancers) or distal colon cancer (descending, sigmoid, and rectal cancers) by the location of CRC.

In all, 60 of the 78 patients with CRC underwent curative or palliative surgical resection of their primary lesions. Tumour surface area was calculated by multiplying the maximum diameter by the diameter crossing at the angle by circle ratio (Table 1).

### RNA isolation

RNA was isolated from faecal samples by using a previously published method (Kanaoka *et al*, 2004; Takai *et al*, 2009). Briefly, 3 ml of Isogen (Nippon Gene, Tokyo, Japan) was added to sterile 5 ml tubes each containing ∼0.5 g of frozen faecal pellets. The pellets were homogenised for a few minutes using a Handy Microhomogenizer (Microtech Niti-on, Chiba, Japan). The slurries were then poured into sterile 1.5 ml tubes, and centrifuged at 12 000 × *g* for 5 min at 4°C. The supernatants were transferred carefully to new sterile 1.5 ml tubes. To each tube was added 0.3 ml Isogen and 0.3 ml chloroform. The tubes were shaken vigorously for 30 s, and then centrifuged at 12 000 × *g* for 15 min at 4°C. The aqueous phase from each tube was removed carefully, avoiding contamination from the interface, and transferred to fresh 1.5 ml tubes. An equal volume of 70% ethanol was added, and the tubes were vortexed vigorously for 30 s. The mixed solution (0.7 ml) was added to an RNeasy minispin column (Qiagen GmbH, Hilden, Germany), and centrifuged at 8000 × *g* for 15 s at room temperature. The remaining steps were performed according to the manufacturer's instructions.

RNA was extracted from biopsy materials, from DH5a *Escherichia coli* cells, as well as from lactobacillus bacteria (effective microorganism 4), using Isogen (Nippon Gene) according to the manufacturer's instructions. Total RNA concentrations were determined using a NanoDrop 1000 (NanoDrop Wilmington, DE, USA), and the RNA samples were stored at −80°C.

### DNA isolation

Human genomic DNA was isolated from the blood of a control patient using Isogen (Nippon Gene), following the manufacturer's instructions. Total DNA concentrations were determined using NanoDrop 1000 (NanoDrop), and the DNA samples were stored at −20°C.

### Reverse transcription

cDNA was synthesised using reverse transcriptase M-MLV (RNaseH^−^; Takara Bio Inc., Otsu, Japan) with 1 *μ*g RNA from faeces or tissue and 250 *μ*g random hexamers, according to the manufacturer's instructions. The product was then amplified using quantitative real-time PCR.

### Quantitative real-time PCR

The gene expression analysis of total isolated RNA focused on selected genes: COX-2, B2M, CD45, CEA, and E-cad. Commercially available TaqMan primers and probe mixtures (Applied Biosystems, Foster City, CA, USA) were used for all reactions. FAM was used as the reporter dye at the 5′-end of probes, and minor grove binder was used as the quencher dye at the 3′-end. The reaction mixture comprised 10 *μ*l of TaqMan Fast Universal PCR Master Mix (Applied Biosystems), 1 *μ*l of 20 × TaqMan primers and probe mixture and 1 *μ*l of template cDNA or genomic DNA in a total reaction volume of 20 *μ*l. Each PCR was performed with precycling heat activation at 95°C for 20 s, followed by 60 cycles of denaturation at 95°C for 3 s, and annealing and extension at 62°C for 30 s in an Applied Biosystems 7500 Fast Real-Time PCR system.

A standard reference curve was established for each marker using serial 10-fold dilutions of the recombinant plasmid DNA containing the target sequence, which was generated using the TOPO TA cloning kit (Invitrogen, Carlsbad, CA, USA) following the manufacturer's instructions. Each sample was run in triplicate and a negative control without template was also run in each reaction plate.

### Statistical analysis

The differences in the faecal expression of target genes between CRC patients and control subjects were analysed by the Mann–Whitney *U*-test. Correlations between COX-2 mRNA expression in faeces and that of other genes, as well as between COX-2 and CEA mRNA expression in faeces and COX-2 and CEA mRNA expression in cancer tissues, were determined from Spearman's rank correlation. The correlation between CEA mRNA expression in faeces and serum CEA was also determined from Spearman's rank correlation. All statistical tests were two-sided, and *P* values <0.05 were considered to be statistically significant.

## Results

### Specificity of PCR assays

In our assay, faecal RNA was isolated without separating cell components from faeces. Therefore, RNAs isolated using our method contain RNAs from human cells and intestinal flora. To confirm that the mRNA evaluated in this study originated from human cells, the specificity of PCR assays for total target gene expressions was investigated using bacterial cDNA, human genomic DNA, RNA isolated from faeces, and cDNA derived from normal colon. As amplification was only observed in the cDNA from normal colon (data not shown), all PCR assays in this study were apparently specific for human cDNA.

### COX-2, B2M, CEA, and E-cad mRNA expression in faeces

**Figure 1:**
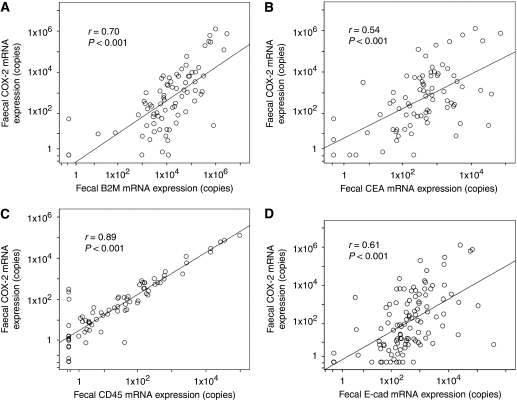
Correlation between expressions of COX-2 mRNA and other mRNAs in faeces. (**A**) Correlation between faecal COX-2 and B2M mRNA expressions in CRC. (**B**) Correlation between faecal COX-2 and CEA mRNA expressions in CRC. (**C**) Correlation between faecal COX-2 and CD45 mRNA expressions in CRC. (**D**) Correlation between faecal COX-2 and E-cad mRNA expressions in CRC. Spearman's rank correlation analysis was applied to determine the correlation.

The median level of faecal COX-2 mRNA expression in CRC patients was significantly higher than that in control subjects (*P*<0.001, Table 2). The median levels of faecal B2M mRNA (*P*<0.05), CEA mRNA (*P*<0.001), and E-cad mRNA (*P*<0.001, Table 2) were also significantly higher in CRC patients than in control subjects. For faecal samples from CRC patients, COX-2 mRNA expression was significantly correlated with B2M mRNA (*r*=0.70, *P*<0.001, Figure 1A), CEA mRNA (*r*=0.54, *P*<0.001, Figure 1B), and E-cad mRNA (*r*=0.61, *P*<0.001, Figure 1D) expressions, suggesting that the increased faecal expression of COX-2 mRNA is related to the number of exfoliated cells. There was a significant correlation between tumour surface area and faecal expression of COX-2 mRNA, B2M mRNA, and CEA mRNA in CRC patients (Table 3). These findings suggest that faecal COX-2 mRNA expression is related to the number of exfoliated cells.

For CRC patients, there was a significant correlation between COX-2 mRNA expression in faeces and COX-2 mRNA expression in cancer tissues (Table 4). A significant correlation was found between faecal expression of COX-2 mRNA and the product of tumour surface area and tissue expression of COX-2 mRNA. However, there was no significant correlation between CEA mRNA expression in faeces and CEA mRNA expression in cancer tissues (*r*=−0.12, *P*=0.42) or between the product of tumour surface area and tissue expression of CEA mRNA (*r*=−0.036, *P*=0.81). A significant correlation between faecal expression and expression in tumour tissue suggests that faecal COX-2 mRNA expression originated from tumour tissue. However, the absence of significant correlation between faecal expression and expression in tumour tissue suggests that faecal CEA mRNA expression originated from tumour tissue and normal mucosa.

As serum CEA is a tumour marker of CRC, we evaluated the relationship between CEA mRNA expression in faeces and serum CEA level. There was no significant correlation between them (*r*=−0.083, *P*=0.48).

### CD45 mRNA expression in faeces

Faecal expression of CD45 mRNA was significantly higher in patients with CRC than in controls in which few expressions of CD45 mRNA were found (Table 2). Although the absolute value of CD45 mRNA expression is less when compared with that of COX-2 mRNA expression, the correlation between faecal CD45 and COX-2 mRNA expressions was highly significant (Figure 1C). The faecal expression of CD45 mRNA was also correlated with tumour size (Table 3). These findings suggest that faecal expression of COX-2 mRNA is closely related to inflammatory cell infiltration in tumour lesions.

### Comparison of marker-gene expressions in faeces according to tumour location

As shown in Table 5, there was no significant difference in any marker-gene expression between proximal colon cancer and distal colon cancer (all *P* >0.05). These results indicate that tumour location does not really affect COX-2 mRNA expression in faeces.

## Discussion

The data presented herein show the following: (1) faecal COX-2 mRNA expression is upregulated in patients with CRC, reconfirming our previous findings (Kanaoka *et al*, 2004, 2007; Takai *et al*, 2009), (2) faecal COX-2 mRNA expression is significantly correlated with faecal B2M, CEA, and E-cad mRNA expressions that are upregulated in CRC, (3) faecal COX-2 mRNA expression is also significantly correlated with faecal expression of CD45 mRNA, which is expressed only in patients with CRC, and (4) faecal COX-2 mRNA expression is significantly correlated with tumour size and with COX-2 mRNA expression in tumour tissues, as well as with the product of tumour size and COX-2 mRNA expression in primary tumour.

FOBT is useful for screening CRC lesions in which bleeding occurs easily and has been widely used as a screening test for CRC. The introduction of FOBT in CRC screening reduced the morbidity and mortality associated with CRC (Mandel *et al*, 1993; Hardcastle *et al*, 1996; Scholefield *et al*, 2002; Lindholm *et al*, 2008). However, FOBT is less sensitive for detecting proximal CRC lesions and small lesions (Morikawa *et al*, 2005). We previously reported that faecal COX-2 mRNA assay is useful for detecting CRC (Kanaoka *et al*, 2004, 2007; Takai *et al*, 2009). However, the factors that contribute to faecal COX-2 mRNA expression in CRC have not been evaluated.

As exfoliated cells, which contain cancer cells, are abundant in the mucocellular layer overlying CRC lesions and surrounding mucosa (Ahlquist *et al*, 2000; Loktionov, 2007), increased faecal COX-2 mRNA expression may be caused by the increase in these exfoliated cells. In this study, quantitative RT–PCR for faecal B2M mRNA expression was performed to assess the number of exfoliated cells in faeces, and that for faecal CEA and E-cad mRNAs expression was performed to assess the number of exfoliated colonocytes. The faecal expressions of B2M, CEA, and E-cad mRNAs in CRC patients were significantly higher than those in control subjects, confirming that exfoliated cells were quantitatively abundant in CRC. The increase in exfoliated cells in CRC does not directly mean an increase in the exfoliation of COX-2 mRNA-containing cells, as the exfoliated non-malignant colonocyte does not express COX-2 mRNA. However, the significant relations between faecal COX-2 mRNA and B2M, CEA, or E-cad mRNAs suggest that increased number of exfoliated cells may contribute to the increase in the faecal expression of COX-2 mRNA. The significant correlation between tumour size and the faecal expression of B2M or CEA mRNAs supports the idea that the increase in exfoliated cells in CRC originated from tumour or surrounding tissues. The increase in faecal CD45 mRNA expression in CRC patients also support the hypothesis that exfoliated cells are increased in CRC, as the mucocellular layer overlying CRC lesions was reported to contain inflammatory cells (Ahlquist *et al*, 2000). As it is well known that inflammation promotes CRC (Balkwill and Mantovani, 2001) and that leukocyte infiltration is often observed within CRC tissue or surrounding tissue, the increase in CD45 mRNA expression in faeces may reflect leukocyte infiltration into colonic mucosa. This was supported by the fact that faecal CD45 mRNA expression was only observed in patients with CRC and was significantly correlated with tumour size. There was a strong correlation between CD45 mRNA expression and COX-2 mRNA expression, suggesting that faecal COX-2 mRNA expression is affected by inflammatory cell infiltration in tumour tissue. The strong correlation between faecal expressions of COX-2 and CD45 mRNAs may be caused by the involvement of inflammation in the development of CRC. As inflammatory cells are known to express COX-2 (Sano *et al*, 1995), it is also possible that the close correlation between faecal COX-2 and CD45 mRNA expressions was caused by the co-expression of COX-2 and CD45 mRNAs by inflammatory cells. Since ulcerative colitis or acute inflammatory condition is susceptible to an increased number of exfoliated epithelial and inflammatory cells, faecal COX-2 mRNA expression is probably elevated in such inflammatory conditions. It is very important for faecal COX-2 mRNA assay to verify whether it could discriminate CRC from other gastrointestinal pathologies such as inflammatory bowel conditions. We need to clarify this issue for future studies.

It is well known that COX-2 is upregulated in not all CRC but most CRC (Kawasaki *et al*, 2008). Therefore, COX-2 mRNA expression in faeces may be affected by the degree of COX-2 mRNA expression in cancer tissues and by tumour size. In this study, a significant relationship between faecal COX-2 mRNA expression and COX-2 mRNA expression in cancer tissues or tumour size was found. When we evaluated the relationship between COX-2 mRNA expression in faeces and the product of COX-2 mRNA expression in cancer tissue and tumour size, a higher correlation was observed between them. This close relationship in COX-2 mRNA expressions between cancer tissue and faeces indicated that faecal COX-2 mRNA originated from neoplastic lesion. These results suggested that COX-2 mRNA expression in cancer tissue and tumour size significantly contributed to COX-2 mRNA expression in faeces.

FOBT is reported to be less sensitive to proximal CRC than to distal CRC (Morikawa *et al*, 2005). To evaluate the contribution of tumour location to faecal COX-2 mRNA expression, we compared the COX-2 mRNA expression in faeces between proximal CRC and distal CRC. There was no significant difference in faecal COX-2 mRNA expression between the groups, suggesting that faecal COX-2 mRNA assay may be useful for detecting proximal CRC and distal CRC.

In conclusion, the results of this study suggest that faecal COX-2 mRNA expression in CRC originated from tumour or surrounding mucosa. The amount of faecal COX-2 mRNA expression was affected by multiple factors, including an increased number of exfoliated cells from tumour, exfoliation of inflammatory cells, tumour size, and COX-2 mRNA expression in tumour tissue, however, not by tumour location.

## Figures and Tables

**Table 1:** Clinical characteristics of the CRC patients and control subjects

	**CRC (*n*=78)**	**Control (*n*=36)**
Age (years), median (range)	68 (48–86)	65 (20–85)
		
*Sex*		
Female:male	24:54	14:22
		
*Tumour site*		
Proximal colon cancer	26	
Distal colon cancer	52	
Tumour surface area (cm^2^), median (range)	51 (3–307)	
		
*Stage*		
0	5	
I	14	
II	31	
III	15	
IV	13	

**Table 2:** Marker-gene expressions in faeces

	**Control**	**CRC**	***P***
COX-2 mRNA (copies) median (range)	5 (0–48)	4.4 × 10^2^ (0–1.3 × 10^6^)	<0.001
B2 M mRNA (copies)	7.0 × 10^3^ (0–1.8 × 10^5^)	1.2 × 10^4^ (0–2.9 × 10^6^)	0.024
CEA mRNA (copies)	1.0 × 10^2^ (0–1.1 × 10^4^)	4.4 × 10^2^ (0–7.0 × 10^4^)	<0.001
E-cad mRNA (copies)	1.7 × 10^2^ (0–2.3 × 10^3^)	6.3 × 10^2^ (0–3.7 × 10^5^)	<0.001
CD45 mRNA (copies)	0 (0–4)	25.0 (0–9.7 × 10^4^)	<0.001

*P* value was analysed by Mann–Whitney test; *P*<0.05 was considered statistically significant.

**Table 3:** Correlation between tumour surface area and marker-gene expressions in faeces

**Marker**	**Tumour surface area *r***	***P***
COX-2	0.33	0.011
B2M	0.29	0.027
CEA	0.32	0.014
E-cad	0.16	0.22
CD45	0.37	0.004

Spearman's rank correlation analysis was applied to determine the correlation; *P*<0.05 was considered statistically significant.

**Table 4:** Correlation between COX-2 mRNA expression in faeces and the product of COX-2 mRNA expression in cancer tissue and tumour surface area

	**COX-2 mRNA expression in faeces**	***P***
COX-2 mRNA expression in cancer tissue	0.38	0.012
COX-2 mRNA expression in cancer tissue × tumour surface area	0.60	<0.001

Spearman's rank correlation analysis was applied to determine the correlation; *P*<0.05 was considered statistically significant.

**Table 5:** Marker-gene expressions in faeces according to tumour location

	**Proximal colon cancer (*n*=26)**	**Distal colon cancer (*n*=52)**	***P***
COX-2 mRNA (copies) median (range)	5.3 × 10^2^ (0–7.5 × 10^5^)	3.1 × 10^2^ (0–1.3 × 10^6^)	0.53
B2 M mRNA (copies)	1.1 × 10^4^ (0–2.9 × 10^6^)	1.6 × 10^4^ (15–9.8 × 10^5^)	0.58
CEA mRNA (copies)	5.0 × 10^2^ (0–7.0 × 10^4^)	3.8 × 10^2^ (2.0–3.9 × 10^4^)	0.68
E-cad mRNA (copies)	7.1 × 10^2^ (0–1.1 × 10^5^)	5.2 × 10^2^ (0–3.7 × 10^5^)	0.46
CD45 mRNA (copies)	20 (0–3.5 × 10^4^)	35.0 (0–9.7 × 10^4^)	0.69

*P* value was analysed by Mann–Whitney test; *P*<0.05 was considered statistically significant.
